# Computer Aided Drug Design Approach to Screen Phytoconstituents of *Adhatoda vasica* as Potential Inhibitors of SARS-CoV-2 Main Protease Enzyme

**DOI:** 10.3390/life12020315

**Published:** 2022-02-20

**Authors:** Bathula Siva Kumar, Singh Anuragh, Ananth Kumar Kammala, Kaliappan Ilango

**Affiliations:** 1 Department of Pharmaceutical Chemistry, SRM College of Pharmacy, SRM Institute of Science and Technology, Kattankulathur, Chengalpattu 603203, Tamil Nadu, India; bk3467@srmist.edu.in; 2 Department of Pharmacology, SRM College of Pharmacy, SRM Institute of Science and Technology, Kattankulathur, Chengalpattu 603203, Tamil Nadu, India; anuragh199522@gmail.com; 3 Department of Obstetrics and Gynecology, The University of Texas Medical Branch, Galveston, TX 77555, USA; ankammal@utmb.edu

**Keywords:** SARS-CoV-2, COVID-19, *Adhatoda vasica*, molecular docking, molecular dynamic simulations

## Abstract

A novel coronavirus (COVID-19) was identified as one of the severe acute respiratory syndrome coronaviruses (SARS-CoV-2) and emerged as a pandemic in 2020. Thus, there is an urgent need to screen and develop an agent to suppress the proliferation of viral particles of SARS-CoV-2, and several drugs have entered clinical trial phases to assess their therapeutic potential. The objective of the present study is to screen phytochemicals against the main viral protease using molecular docking studies. The phytochemicals vasicine, vasicinone, vasicinolone, vasicol, vasicolinone, adhatodine, adhavasicinone, aniflorine, anisotine, vasnetine, and orientin from *Adhatoda vasica* were selected, and the compounds were docked with various viral protein targets, including specific SARS-CoV-2 main protease (PDBID:6Y84), using AutoDock, Schrodinger, Biovia discovery studio, and virtual screening tools. Adhatodine and vasnetine showed a better binding affinity of −9.60 KJ/mol and −8.78 KJ/mol, respectively. In molecular docking simulations for 10 ns, these compounds illustrated strong hydrogen-bonding interactions with the protein active site and induced a potential conformational change in the ligand-binding site. The results were compared with the antiviral drugs nirmatrelvir and ritonavir. These results suggest that these phytochemicals can be studied as potential inhibitors against SARS-CoV-2 protease and may have an antiviral effect on coronavirus. However, further in vitro and in vivo efficacy activity needs to be investigated for these phytochemicals.

## 1. Introduction

Severe acute respiratory syndrome coronavirus-2 (SARS-CoV-2), also known as COVID-19, is a unique strain of coronavirus that initially spread from the city of Wuhan, China, although its actual origin is unknown [1]. A viral epidemic emerged in 2003 as a severe acute respiratory syndrome (SARS) in China [2]. COVID-19 has emerged as a contagious respiratory illness that has chiefly affected 215 countries; as of 27 January 2022, there had been more than 360,578,392 cumulative cases and 5,620,865 cumulative deaths [3]. The symptoms of cold, fever, dry cough, fatigue, shortness of breath, and loss of smell and taste are commonly observed in COVID-19 affected patients [4]. Coronaviruses have already infected humans in the form of SARS-CoV and Middle East respiratory syndrome coronavirus (MERS-CoV). SARS-CoV-2, which belongs to the betacoronavirus genus, causes severe to deadly pneumonia in humans [5]. A growing body of literature indicates that immunological patterns are intimately linked to the course of illness in people infected with viruses. Reducing peripheral T cell subsets is a distinct feature of SARS patients [6].

Coronavirus spike proteins help the virus bind to receptors and infiltrate host cells. Although the spike protein is essential for receptor-mediated viral replication, it is also helpful as an immunogen, since it is the most exposed component of the viral anatomy. The structural proteins of SARS-CoV-2 or COVID-19 are N (nucleocapsid), M (membrane), E (envelope), and S (structural protein) (spike). Although these proteins are involved in the viral formation, only E, M, and S can be seen on the virus particle’s outer surface, and the S protein creates a knob-like architecture that is larger than the other structural proteins [7].

Indian people consume traditional medicinal herb extracts and Indian spices to boost the immune system to fight COVID-19 [8]. Adhatoda, also known as Adosa, belongs to the Acanthaceae family and is commonly found in many parts of India and worldwide. It is also widely referred to as the Malabar nut and as Vasaka in Sanskrit, and is well known in the Unani and Ayurvedic classes of medicine [9]. For over 2000 years, this herb has been employed in India’s traditional system of medicine to cure a variety of illnesses and problems, including respiratory tract issues, cough, and colds. The leaves and flowers of the plants are used as vegetables.

**Figure 1:**
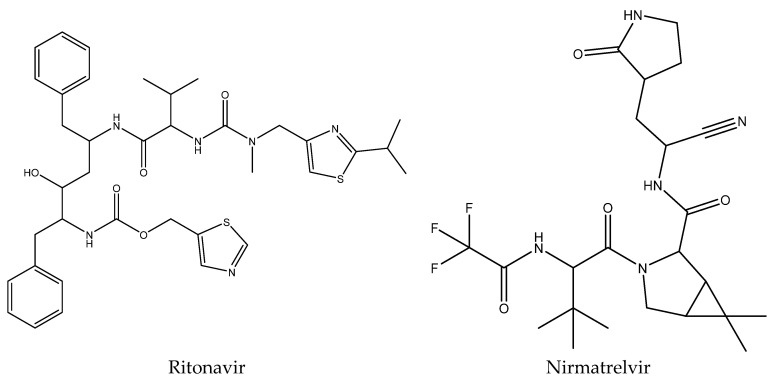
Some of the earlier-known drugs that have been used against SARS-CoV-2.

The aim of the current study was to discover a promising antiviral agent from natural resources [10,11]. In a recent clinical trial, according to an interim analysis of phase 2 and 3 data involving 1219 patients, the probability of COVID-19-related hospital admission or all-cause death was reduced by 89% in the nirmatrelvir (PF-07321332) group when taken within three days of symptom onset, compared to placebo. More studies are being conducted to verify the efficacy evidence presented (Figure 1) [12]. Although several nations employed nirmatrelvir as a COVID-19 treatment therapy during the onset of the pandemic, its use has reduced as vaccines have been developed. In addition, numerous additional medications, both recognized and new, are being tested in clinical trials against SARS-CoV-2, including indinavir, saquinavir, darunavir, ASC09, and ritonavir (Figure 1) [13,14].

## 2. Materials and Methods

The most effective techniques for predicting drug interactions with macromolecules are molecular docking approaches. The blind docking approach entails searching for binding sites across the entire surface of the macromolecule. As a result, blind molecular docking investigations were performed using particular existing medications, as well as some potent bioactive compounds, and assayed using COVID-19′s primary protease, SARS-CoV-2 [15].

### 2.1. Ligand Generation

**Figure 2:**
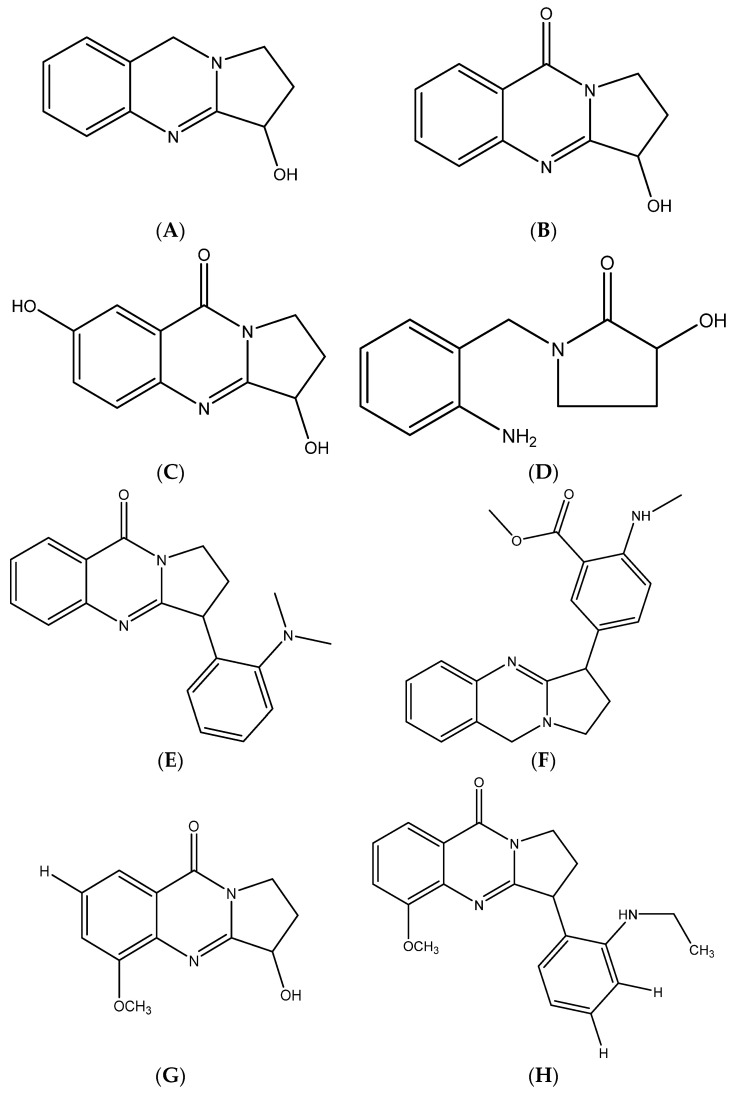

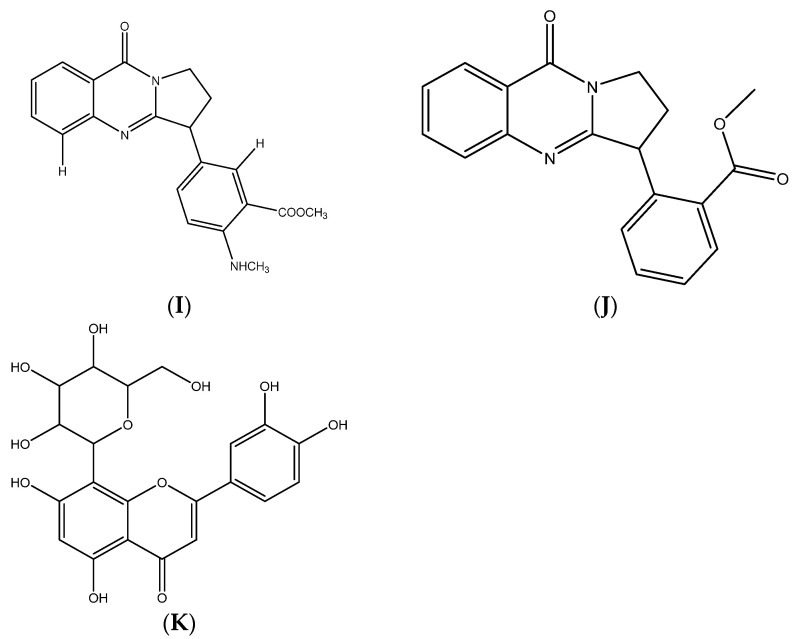
Chemical structures of (**A**) vasicine, (**B**) vasicinone, (**C**) vasicinolone, (**D**) vasicol, (**E**) vasicolinone, (**F**) adhatodine, (**G**) adhavasicinone, (**H**) aniflorine, (**I**) anisotine, (**J**) vasnetine, and (**K**) orientin.

The 2D structure of the phytocompounds of *Adhatoda vasica* (vasicine, vasicinone, vasicinolone, vasicol, vasicolinone, adhatodine, adhavasicinone, aniflorine, anisotine, vasnetine, and orientin) were retrieved from the PubChem database of chemical molecules [16]. The retrieved 2D.sdf file format was converted into a 3D SDF format using the SMILES converter and structure file generator (Figure 2) [17].

### 2.2. Drug Likeliness and ADME and Toxicity Calculations

The SwissADME online server was used to calculate the drug likeliness parameters. The drug likeliness of the *Adhatoda Vasica* phytocompounds (vasicine, vasicinone, vasicinolone, vasicol, vasicolinone, adhatodine, adhavasicinone, aniflorine, anisotine, vasnetine, and orientin) was based on violations of the following rules: Lipinski, Ghose, Veber, Egan, and Muegge. Pre-ADMET, a web-based application, was used to determine the pharmacological efficiency of the pharmacokinetic properties of the 11 screened phytocompounds of *Adhatoda vasica* [18].

### 2.3. Preparation of Receptors and Their Binding Site

The core viral molecules involved in viral particle attachment, replication, and reproduction in host cells include proteases, the spike protein, the RNA binding protein, N-terminal RNA binding domain. These protein target molecules were identified as a new scapegoat for inhibiting the viral life cycle in human host cells. The RCSB PDB Database was used to generate the three-dimensional structure of the SARS-CoV-2 main protease [6Y84] [19]. The amino acids with binding pockets were used to measure the binding affinities between both the ligand and the receptor.

### 2.4. Molecular Docking

Using AutoDock, the produced Adhatoda SDF Structures were docked with the anticipated binding site of all specified protein target binding sites [20]. This is a well-known dynamic docking methodology, with the following parameters:

#### 2.4.1. Protein Preparation

The protein was used to predict the behavior of the mentioned compounds against the macromolecular targets of the human coronavirus (PDB 6Y84). For the purpose of crystal structure energy minimization, we utilized the Swiss-PDB Viewer software package and then removed all the heteroatoms and water molecules of the proteins using Biovia discovery studio. This was followed by H-bond optimization and the addition of Kollmann charges, which were carried out for the protein preparation protocol and finally saved to pdbqt charges.

#### 2.4.2. Active Site and Grid Generation

The MMFF94 force field was utilized in 2000 steps, with a Van der Waals scaling factor of 1.00 and a 0.25 charge cut off. The bounding box for the docking investigation was a 60 × 60 × 60 Å grid, defined and employed with a grid spacing of 0.534 Å. Docking and critical analysis are two terms that have been used to describe the process of docking and binding. In the original target protein grids, the active sites were calculated by the Drug Discovery Studio version and used as the coordinates of the ligand in that grid (BIOVIA Dassault Systems). To determine the non-covalent interactions, the researcher utilized Discovery Studios Software [21].

#### 2.4.3. Genetic Algorithm

This is a heuristic for screening, based on Charles Darwin’s theory of natural selection. This algorithm is based on the evolutionary process, in which the fittest individuals are selected for procreation, to ensure future posterity.

#### 2.4.4. Lamarckian Genetic Algorithm

We constructed a Lamarckian genetic algorithm using Solis and Wets’ local search technique to compare our novel approach to a genetic algorithm that was comparable to the one used in the well-known docking package AutoDock 4.2.6. The Solis and Wets technique is a stochastic heuristic for continuous parameter spaces.

#### 2.4.5. Ligand–Receptor Interactions

The interactions of *Adhatoda vasica* phytocompounds with SARS-CoV-2 protein targets in the docked complex were analyzed by the pose view of Lead [22]. 2D and 3D Pose view of protein target phytocompounds *Adhatoda vasica* was generated and investigated its lead.

#### 2.4.6. Molecular Dynamics Simulations

The Schrodinger Desmond package simulated molecular dynamics (MD) on the following complexes. After docking, the two best low binding energy ligand-protein complexes were: (1) free protein; (2) protein co-crystallized with N3 potent inhibitor (we identified a mechanism-based inhibitor (N3) by computer-aided drug design, and then determined the crystal structure of the Mpro of SARS-CoV-2 in complex with this compound); and the top two best low binding energy ligand-protein complexes. Individually, all the compounds were solvated in an explicit water box of size 10, with periodic boundary conditions (PBCs), using a single-point charge (SPC) water model TIP3P. (PBC). The OPLS2005 force field [23,24] was used to represent the protein and ligand, and Na^+^ and Cl^−^ ions were added to make the overall charge of the system neutral. The system was then subjected to a 2000-step energy reduction, before being put through a 10 ns production cycle. After minimization, the complex was put through a manufacturing run at the NPT ensemble. The system was slowly heated to maintain a temperature of 300 K and constant pressure, using the Nose–Hoover thermostatic algorithm and the Martina-Tobias-Klein technique. The particle mesh Ewald (PME) approach was used to compute long-range electrostatic interactions, with a grid spacing of 0.8. The Simulation Interaction Diagram tool contained in the Desmond package was used to study the specific interactions between the ligand and protein. The results were compared to the reference in protein and ligand RMSD and root means square fluctuation (RMSF) values. Pant et al. [25] described a similar approach.

MD simulation is a computer method for predicting molecular variability by plotting trajectories. The best posed bound complexes with the most potent binding energies from the molecular docking research were recovered independently and subjected to MD simulation analysis using Schrodinger Desmond. Before going on to MD simulation, the topology and gro file for the chosen drug was created on PRODRUG (an online service), and then the OPLS2005 force field was used to build the protein–ligand complex [26,27]. They were then subjected to energy minimization, following the addition of ions. After energy minimization, NVT (volume regulation) and NPT (pressure regulation) were conducted to complete the system’s equilibration. Finally, a ten nanosecond MD run was employed, using a leap-frog integrator with a step size of 2 fs in the total MD run. The data were stored every two picoseconds for stability analysis [28].

## 3. Results and Discussion

### 3.1. Drug Likeliness Prediction [ADME] Calculations

The Lipinski’s rule of five criteria (molecular weight less than 500 Da, no more than five hydrogen bond donors, no more than ten hydrogen bond acceptors, rotatable bonds less than 10, molar refractivity 40–130, and logP less than 5) indicates a molecule’s drug likeliness. The SwissADME server was used to calculate drug likeliness for the *Adhatoda vasica* phytocompounds (vasicine, vasicinone, vasicinolone, vasicol, vasicolinone, adhatodine, adhavasicinone, aniflorine, anisotine, vasnetine, and orientin). There are no breaches for drug likeliness rules such as Lipinski, Ghose, Veber, Egan, and Muegge, which is essential for enhanced drug likeliness features (Table 1) [26].

### 3.2. Docking Study

In Table 2, different COVID-19 target proteins (proteases, spike proteins) are listed, along with their docking score and 3D pose with *Adhatoda vasica* phytocompounds (vasicine, vasicinone, vasicinolone, vasicol, vasicolinone, adhatodine, adhavasicinone, aniflorine, anisotine, vasnetine, and orientin), as well as their detailed molecular interaction.

**Figure 3:**
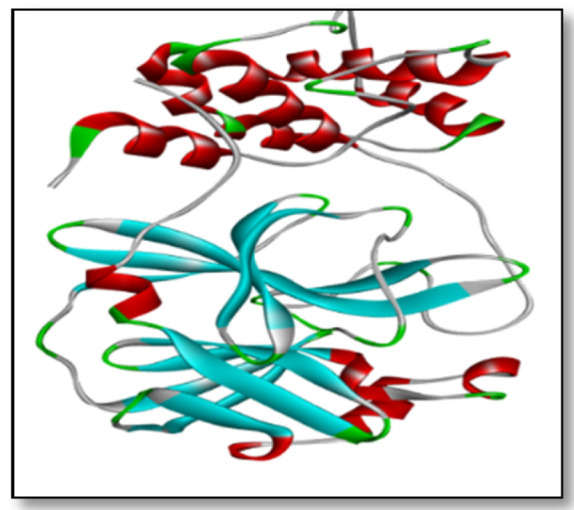
The co-crystal structure of replicase polyprotein.

The Protein Data Bank (PDB) (Figure 3) yielded the crystal structure of replicase polyprotein PDB ID: [6Y84]. The final design was a dimer comprising two homologous amino acid chains, with chain A serving as the molecular docking component.

The AutoDock software docking analysis findings between COVID-19 viral targets and *Adhatoda vasica* revealed binding affinity and docking scores ranging from −6.06 KJ/mol to −9.60 KJ/mol, with adhatodine having the most significant binding relationship with 6Y84, at −9.60 KJ/mol. The major protease of COVID-19 is required for SARS-CoV-2 replication and reproduction. PHE-8, PRO-9, GLN-127, and ASP-295 are coronavirus viral protease residues resulting from hydrogen-bonded interactions with *Adhatoda vasica* phytocompounds.

**Figure 4:**
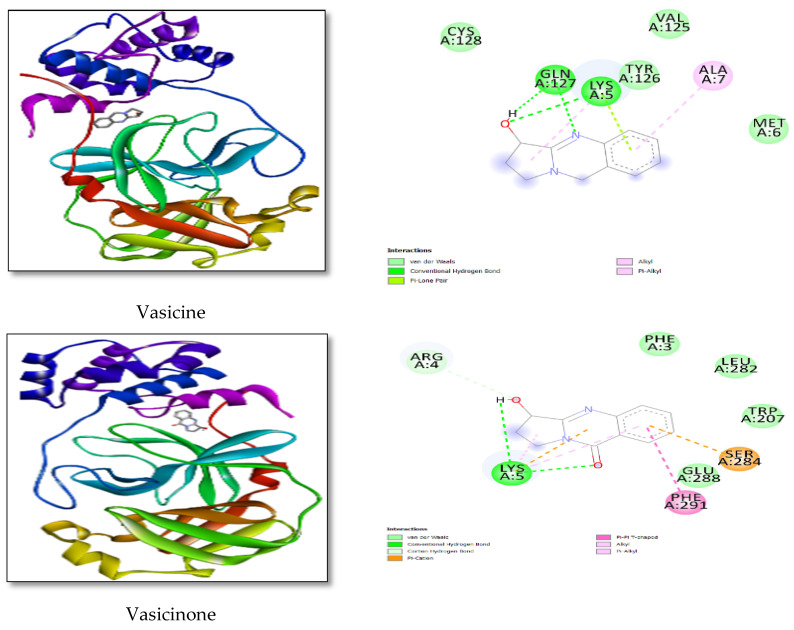

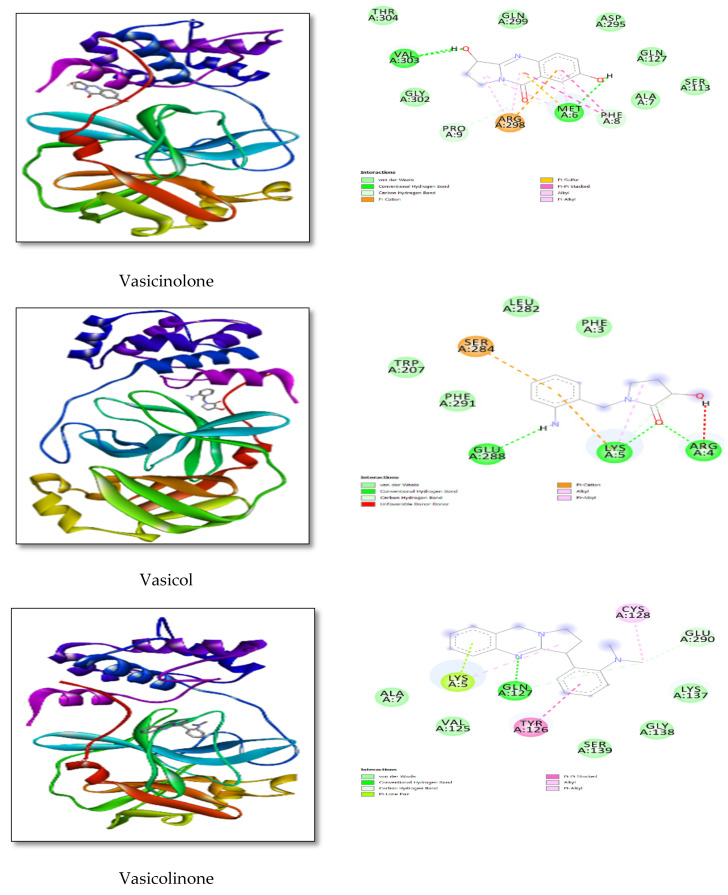

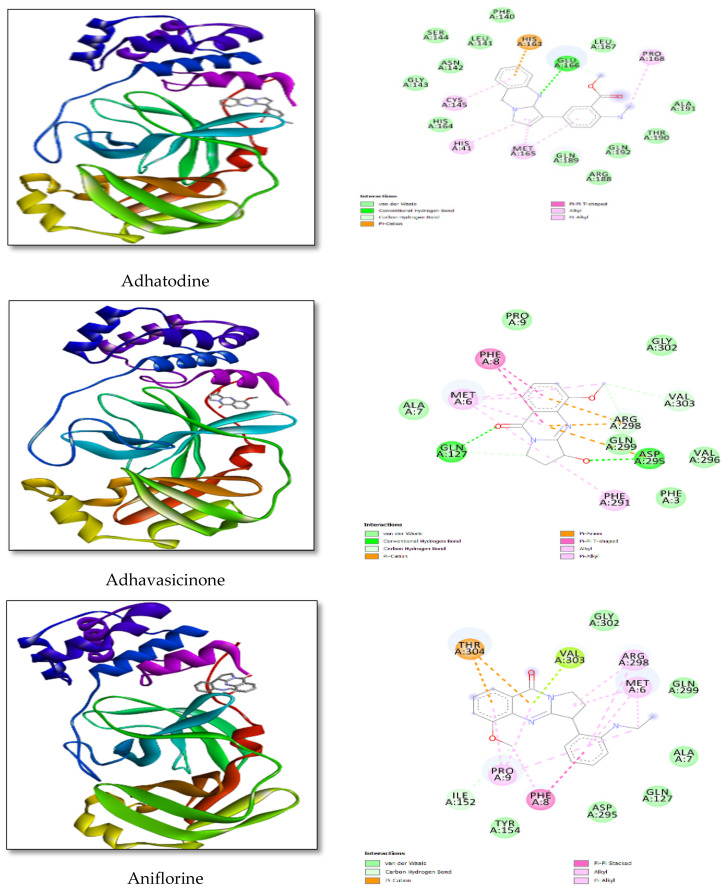

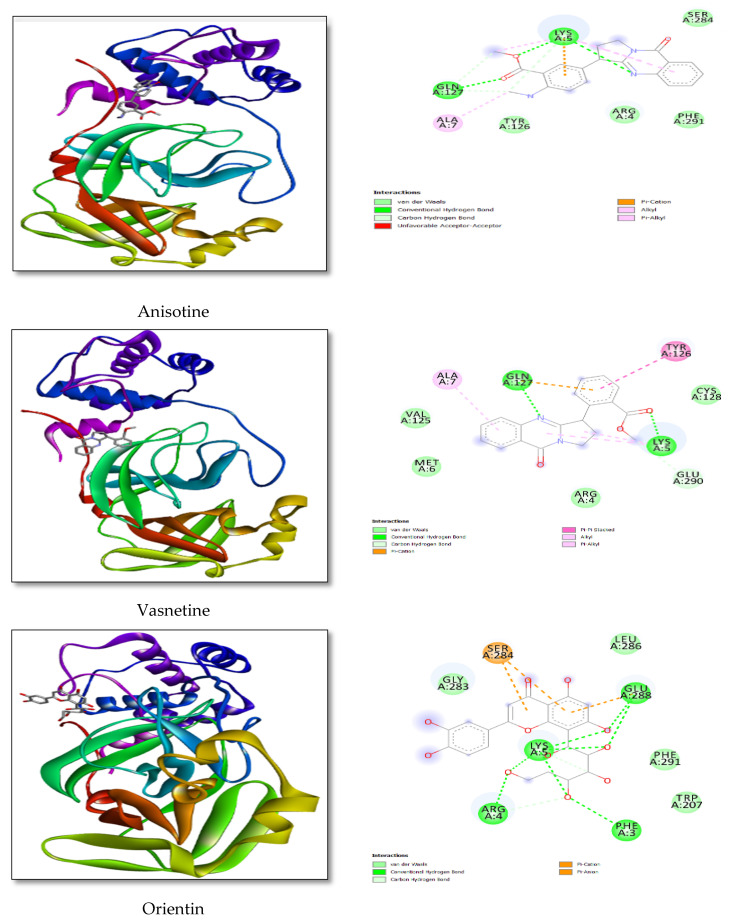
The minimum docked poses of chemical compounds and their corresponding 2 D interaction plots within the active site of SARS-CoV-2.

According to docking findings, the drug binds at the protein’s active region, engaging with critical residues via polar and non-polar interactions, and the existence of an H-bond was detected with Vasicine LYS5, and GLN127, as well as hydrophobic interactions with MET6, ALA7, and Figure 4 shows the docking posture and ligand interaction diagram. The minimum binding energy for this interaction was found to be –6.90 kcal/mol. The connection that inhibits the ReP protein will aid in an essential phase in the replication and translation mechanisms.

Vasicinone interactions with LYS5 were also seen in the presence of an H-bond, as were hydrophobic interactions with PRO9, GLN127, and ASP295; the interaction between the docking posture and the ligand was determined to be −7.19 kcal/mol. Vasicinolone interactions with MET6, VAL303, and hydrophobic interactions with LYS5, TRP207, and LEU282, were found; the interaction between the docking posture and the ligand was determined to be −6.81 kcal/mol. Vasicol interactions with LYS5, ARG4, GLU288, and hydrophobic interactions with PHE8, ILE152, and ARG298 were also detected in the presence of an H-bond; the interaction between the docking posture and the ligand was determined to be −7.19 kcal/mol.

Vasicolinone interactions with GLN127 were detected in the presence of an H-bond, as were hydrophobic interactions with MET6, PHE8, PRO9, ILE152, ARG298, and VAL303; the interaction between the docking posture and the ligand was determined to be −9.06 kcal/mol. Hydrophobic interactions with PRO9, ILE152, GLN127, and ASP295, and adhatodine interactions with GLU166 in the presence of an H-bond were also found; the interaction between the docking posture and the ligand was determined to be −9.60 kcal/mol. Adhavasicinone interactions with GLN127, ASP295, and hydrophobic interactions with PHE8, PHE291, and ASP295 were also identified; the interaction between the docking posture and the ligand was determined to be −6.83 kcal/mol. In hydrophobic contacts with PRO9 and THR309, aniflorine interactions were also seen; the interaction between the docking posture and the ligand was determined to be −6.83 kcal/mol.

Anisotine interactions with LYS5, GLN127, and hydrophobic interactions with LYS5, GLU288, and PHE291 were also found in the presence of an H-bond; the interaction between the docking posture and the ligand was determined to be −8.23 kcal/mol. Vasnetine interactions with LYS5, GLN127, and hydrophobic interactions with LYS5, ALA7, and TYR126 were also found in the presence of H-bonds; the interaction between the docking posture and the ligand was determined to be −8.78 kcal/mol.

In the presence of an H-bond, orientin interactions with PHE3, ARG4, LYS5, and GLU288 were seen, as well as hydrophobic interactions with ARG4, LYS5, and GLU288, the interaction between the docking posture and the ligand was determined to be −8.78 kcal/mol.

These interactions show that replicase polyprotein binds extremely near the active region and inhibits the enzyme very effectively. Figure 4 depicts the docking posture and ligand interaction diagram. The binding energy of this interaction was found to be −9.60 kcal/mol in the lowest energy state. This contact will be critical in preventing RdRp from driving viral genome replication via a complicated mechanism.

Finally, a crucial protein replicates poly protein receptor was the final objective of our research (which is an important first contact of the novel CoV-2). By blocking this receptor, SARS-CoV-2 will be prevented from connecting to the host cell and transferring its genome for replication and translation. When it comes to viral medications, no comparable effects to Rep were noted. The ligand also binds at a similar binding site here, and no active site residues were shown to interact with the ligand. Non-polar interactions were all visible. The ligand-binding was aided by LYS5, LYS137, GLU288, and PHE291. Table 2 shows a docking posture and ligand interaction diagram. The binding energy obtained for remedesivir was −8.87 kcal/mol. The target viral medicines were employed in the viral inhibitor to transit in a closed conformation and attach to the attacking virus.

### 3.3. Molecular Dynamics Simulation

The best-docked findings of adhatodine and vasnetine with SARS-CoV-2 protease (PDB: 6Y84) were submitted to MD simulation tests, utilizing the Desmond 5.6.1 modeling package and Linux as the working environment. Before simulation, PRODRG was used to create specific essential files, such as gro and its files, for the inhibitors adhatodine and vasnetine. The MMFF94 force field was used, then solvation, ion addition, energy minimization, and system equilibration (NVT and NPT) tests were performed. Finally, a 10 nanometer MD simulation was used to create trajectories, before analyzing the final findings regarding RMSD, RMSF, and radius of gyration.

**Figure 5:**
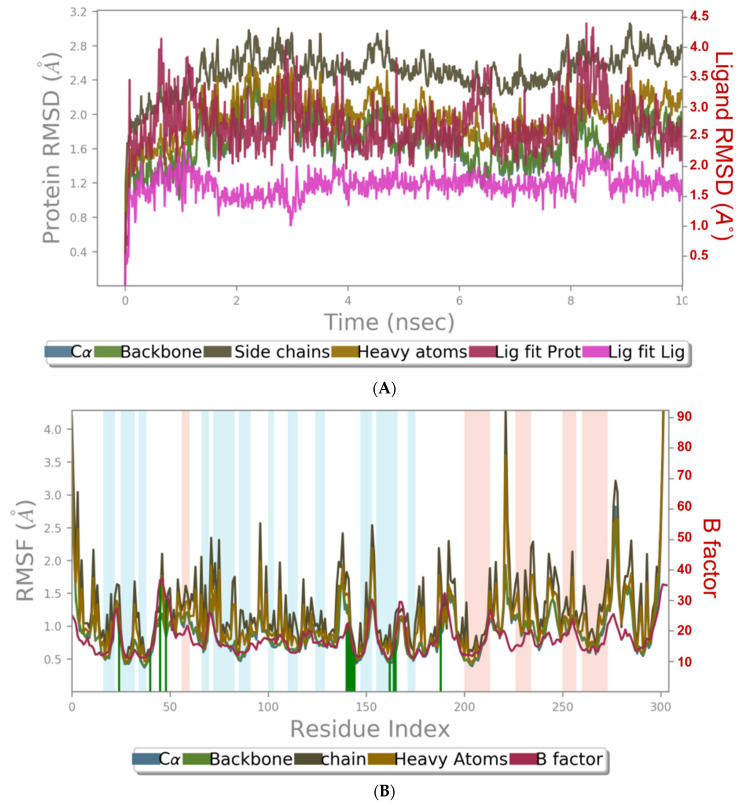
Graphical representation of (**A**) root-mean-square fluctuation for adhatodine with active site residues of 6Y84 after MDS and (**B**) ligand RMSD plot to show the contact point of the ligand with the amino residue of the 6Y84 represented as green color vertical green lines with several contact points.

**Figure 6:**
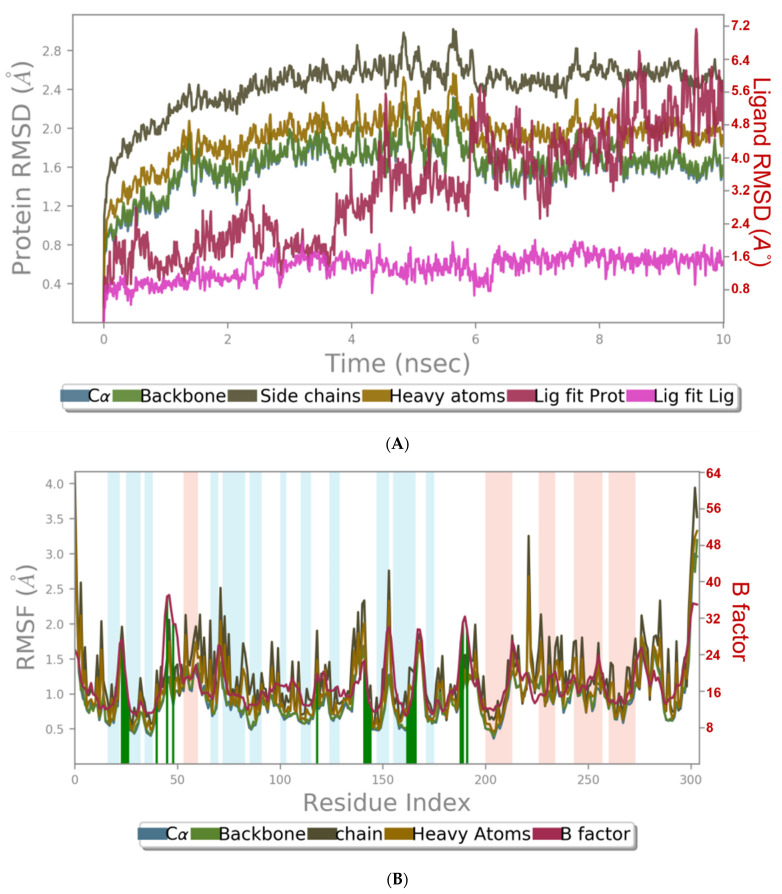
Graphical representation of (**A**) root-mean-square fluctuation for vasnetine with active site residues of 6Y84 after MDS, and (**B**) ligand RMSD plot to show the contact point of the ligand with the amino residue of the 6Y84 represented as green color vertical green lines with several contact points.

**Figure 7:**
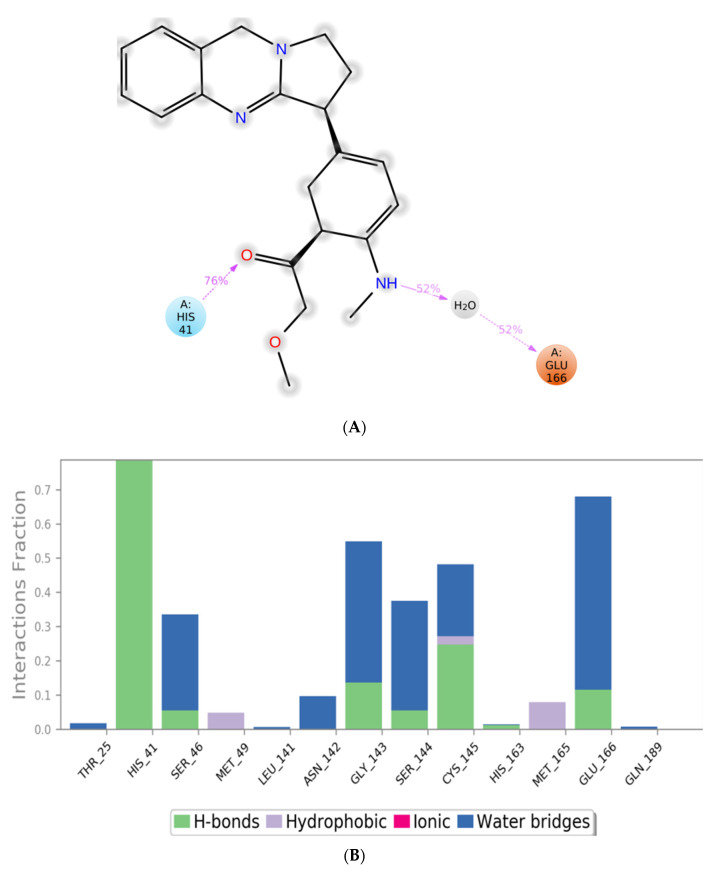

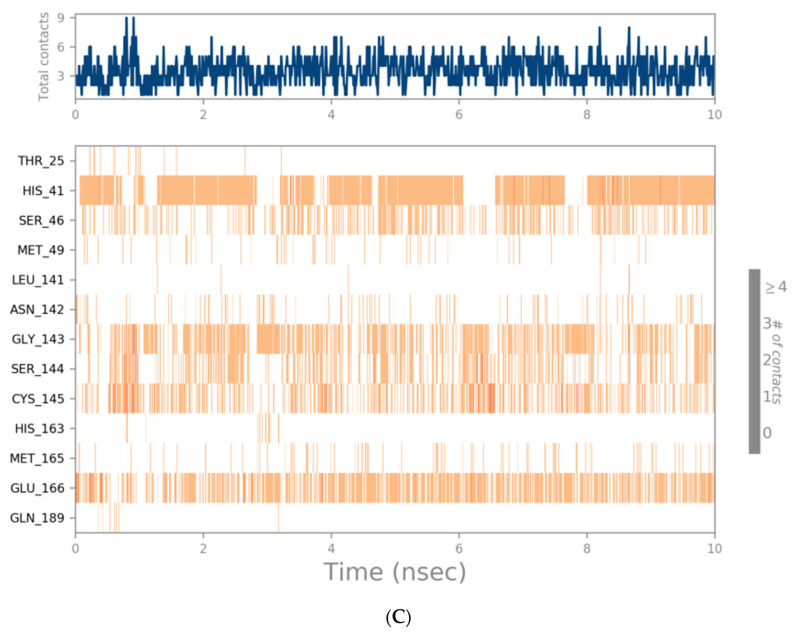
A scheme of detailed adhatodine atom interactions with the protein residues. (**A**) Interactions occur more than 30% of the simulation time in the selected trajectory. (**B**) Normalized stacked bar chart of compound adhatodine interacting with 6Y84 away from active site pocket through a hydrogen bond, hydrophobic and ionic interactions, and water bridges and (**C**) The number of contact points with the amino acid residues are depicted in timeline representations coded with color intensity graphs throughout the simulation.

**Figure 8:**
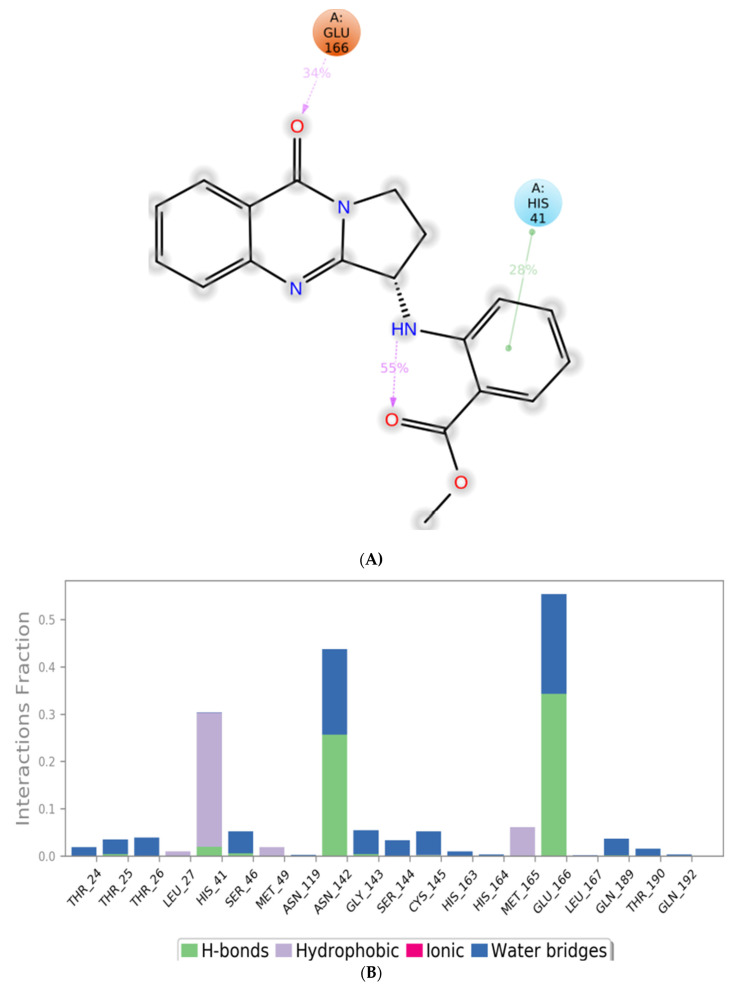

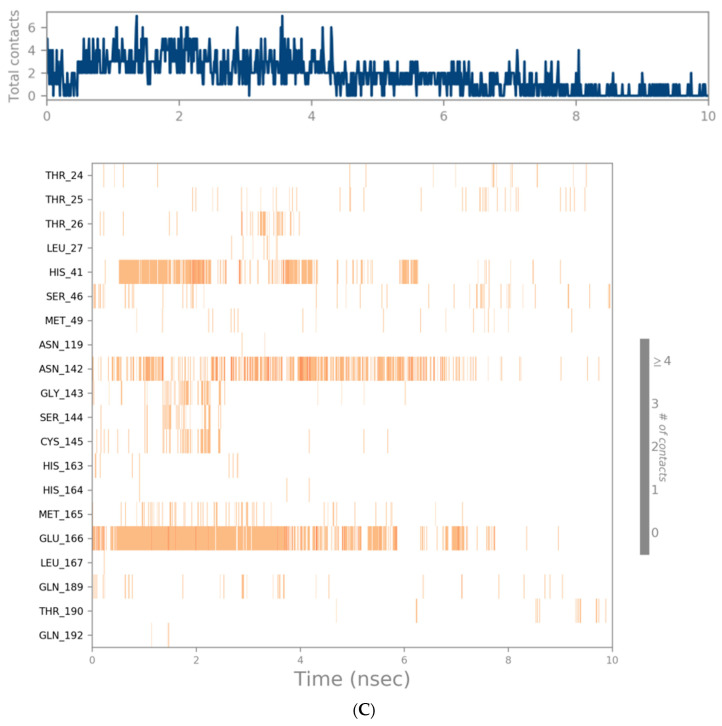
A scheme of detailed vasnetine atom interactions with the protein residues. (**A**) Interactions occur more than 30% of the simulation time in the selected trajectory. (**B**) Normalized stacked bar chart of compound vasnetine interacting with 6Y84 away from active site pocket through a hydrogen bond, hydrophobic and ionic interactions, and water bridges, and (**C**) the number of contact points with the amino acid residues are depicted in timeline representations coded with color intensity graphs throughout the simulation.

**Figure 9:**
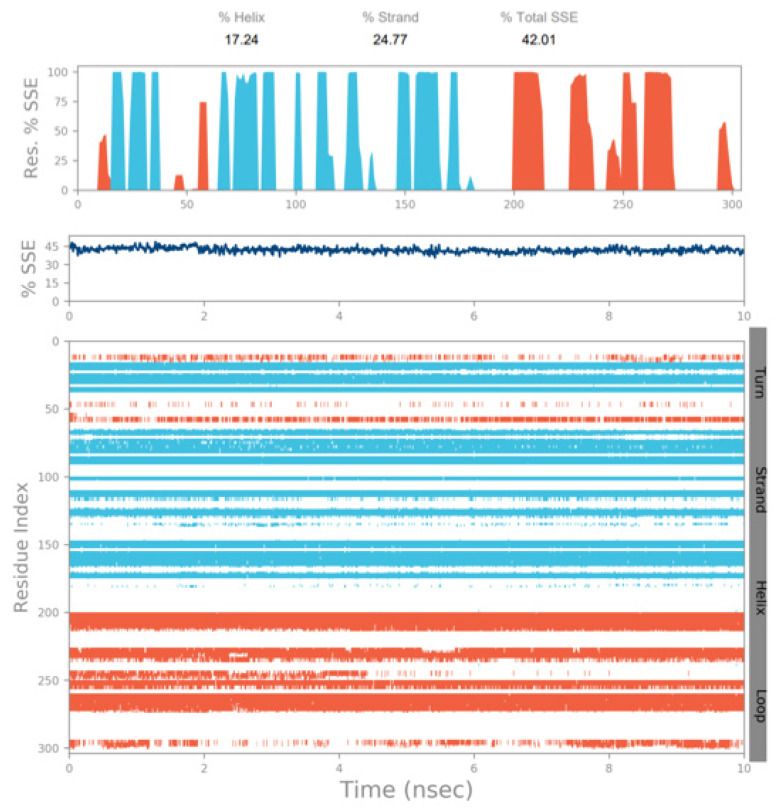
The simulation monitors protein secondary structure elements (SSE) like α-helixes and β-strands are monitored throughout the simulation. The plot above reports SSE distribution by residue index throughout the protein structure for adhatodine.

**Figure 10:**
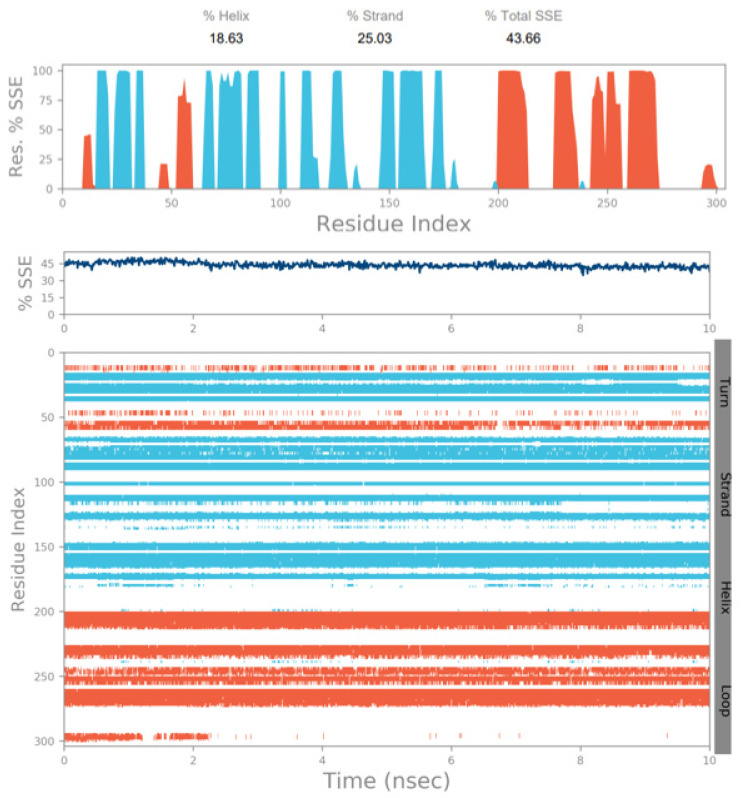
The simulation monitors protein secondary structure elements (SSE) such as α-helixes and β-strands are monitored throughout the simulation. The plot above reports SSE distribution by residue index throughout the protein structure for vasnetine.

**Figure 11:**
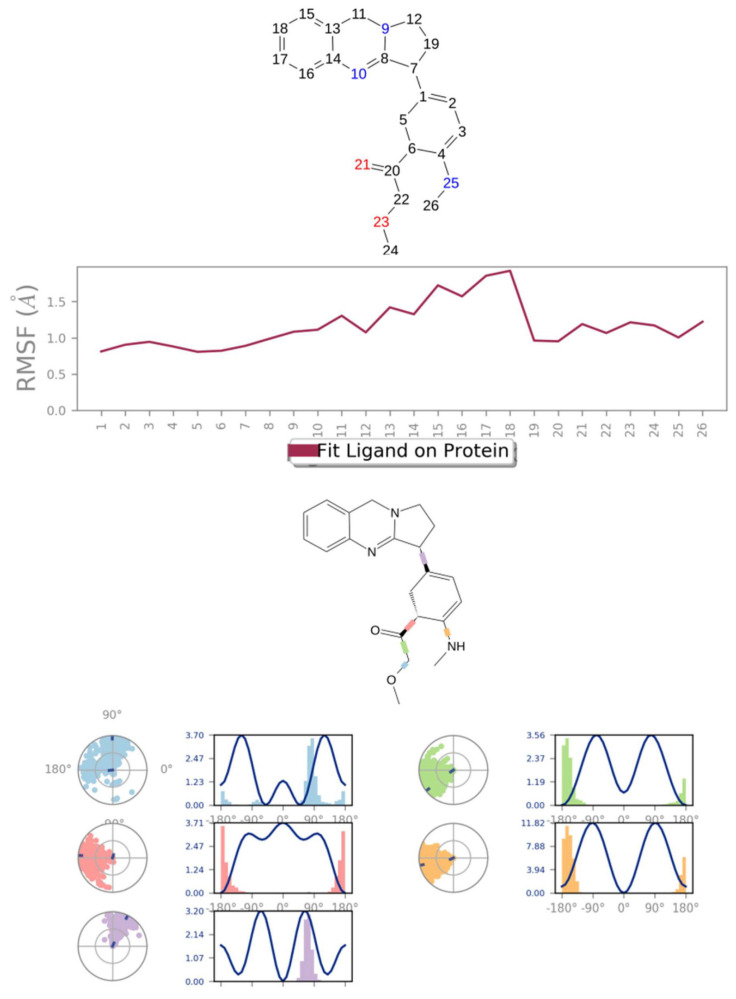
Adhatodine RMSF and torsion profile.

**Figure 12:**
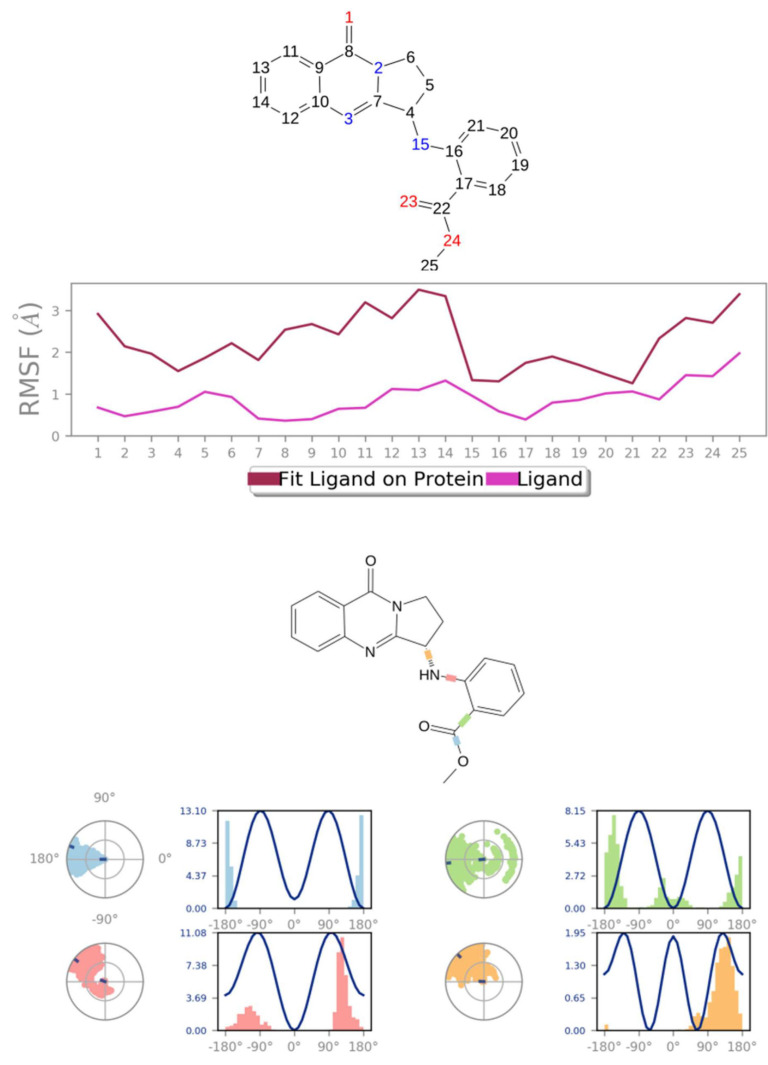
Vasnetine RMSF and torsion profile.

MD simulation experiments were carried out to analyze the protein for the docked protein–ligand complex changes throughout the simulation, to explore the protein dynamics when the adhatodine binds to 6Y84. Desmond equilibrates the system to attain a stable conformation if the initial structure is energetically unstable. MD simulations were conducted on adhatodine, vasnetine, and 6Y84 docked complexes. In the early stages, the RMSD coincided with the ligand RMSD (adhatodine-, vasnetine) and went up to 10 ns. This demonstrates that it created numerous interactions with amino acid residues (Figure 5 and Figure 6). The visual Figure summarizes the interaction and the number of touchpoints. HIS-41 and GLU-166 are essential amino acids that establish interactions and play a vital function in the non-competitive binding site. The adhatodine vasnetine 2D critical geometry with 6Y84 after MD simulation reveals that the adhatodine vasnetine interacts with the optimal binding site of 6Y84 in various ways (Figure 7 and Figure 8). For adhatodine, vasnetine binding to 6Y84, the protein secondary structural elements (SSE) produced 17.24, 24.77, 18.63, and 25.03% alterations in the helix and strands throughout the simulation up to 10 ns (Figure 9 and Figure 10). The graphs below show the SSE composition for each orbital frame during simulation, and the chart at the bottom shows how each residue’s SSE assignment changes over time (Figure 11 and Figure 12). As a result of MD simulation research, it seems that removing adhatodine, vasnetine, and 6Y84 from the ligand-binding site causes a conformational shift in the ligand-binding site.

## 4. Conclusions

COVID-19 is a fast-spreading illness that has resulted in several infections and fatalities across the globe. Lung damage is widespread in COVID-19 individuals, with pulmonary fibrosis occurring in severe instances. Based on the findings of this investigation, out of 11 key *Adhatoda vasica* phytocompounds, it was observed that vasicolinone, adhatodine, aniflorine, anisotine, and vasnetine showed a better binding affinity for the target protein when compared to the standard drug nirmatrelvir, and least affinity was observed with vasicine, vasicinolone, and orientin, when compared to the standard drug ritonavir.

The literature and data repositories were searched for previous reports of active ingredients of *Adhatoda vasica*. From the data on the interactions between the ligands and SARS, molecular docking was undertaken using AutoDock 4.2.6 and the accompanying software Discovery studio 3.5. Enzyme (PDB:6Y84) binding energies of −9.60 KJ/mol and −6.06 KJ/mol were reported for the 11 selected compounds, out of 23 significant compounds. The docking results of the 11 *Adhatoda vasica* compounds were compared with the standard antiviral drugs nirmatrelvir and ritonavir, and the docking score was found to be −8.10 KJ/mol and −7.25 KJ/mol, respectively. Nirmatrelvir is a SARS-CoV-2 main protease inhibitor and works by preventing the growth of the virus causing COVID-19. Ritonavir increases (boosts) the levels of nirmatrelvir, which helps nirmatrelvir to work better in combination. The Lipinski criterion for drug likeliness qualities was met, and the natural substances had little or no adverse effects.

Using the Schrodinger Desmond package, MD simulation analysis was conducted were conducted for ten nanoseconds, to test the stability and flexibility of the protein–ligand complexes. The findings showed that the protein–ligand complexes were stable during the simulation. COVID-19 can be treated with *Adhatoda vasica*, which possesses a promising chemical moiety. The MD simulation findings from the present investigation were acquired for the two phytoconstituents adhatodine and vasnetine; the amino acid residues HIS-41 and GLU-166 were stabilized due to hydrogen bond and hydrophobic interaction. At the non-competitive binding site, they play a crucial function. Many mutant strains of COVID-19 have spread throughout the world, and we can identify specific proteins from those mutant strains and then target those proteins. However, more in vitro and in vivo studies are needed to confirm the activity of individual compounds as antiviral agents against this novel coronavirus.

## Figures and Tables

**Table 1:** Predicted physiochemical properties of the selected compounds.

Comp	MW	iLOGP	HBD	HBA	TPSA	RB	nAH	MR
Vasicine	188.23	1.64	1	2	35.83	-	14	62.11
Vasicinone	202.21	1.61	1	3	55.12	-	15	56.09
Vasicinolone	218.21	1.52	2	4	75.35	-	16	58.11
Vasicol	206.24	1.64	2	2	66.56	2	15	61.10
Vasicolinone	305.37	2.86	-	2	38.13	2	23	93.62
Adhatodine	335.40	3.20	1	3	53.93	4	25	106.2
Adhavasicinone	232.24	1.78	1	4	64.35	1	7	62.58
Aniflorine	335.40	3.05	1	3	56.15	4	25	100.02
Anisotine	349.38	3.21	1	4	73.22	4	26	100.00
Vasnetine	320.34	2.91	-	3	61.19	3	24	90.69
Orientin	448.38	1.27	8	11	201.28	3	32	108.63

**Table 2:** Docking scores of *Adhatoda vasica* phytoconstituents in silico binding with the SARS-CoV-2 main protease (6Y84).

Compounds Name	Binding Affinity (KJ/mol)	Amino Acid-Distance
Vasicine	−6.90	06-MET-3.29
		07-ALA-3.20
		295-ASP-3.59
Vasicinone	−7.19	8-PHE-3.99
		9-PRO-3.30
		127-GLN-3.83
		295-ASP-3.43
Vasicinolone	−6.81	05-LYS-3.68
		207-TRP-3.89
		282-LEU-3.96
Vasicol	−7.19	8-PHE-3.13
		152-ILE-3.72
		298-ARG-3.64
Vasicolinone	−9.06	06-MET-3.65
		08-PHE-3.68
		09-PRO-3.84
		152-ILE-3.65
		298-ARG-3.32
		303-VAL-3.97
Adhatodine	−9.60	8-PHE-3.99
		9-PRO-3.30
		127-GLN-3.83
		295-ASP-3.43
Adhavasicinone	−6.83	8-PHE-3.11
		291-PHE-3.11
		295-ASP-3.02
Aniflorine	−7.78	09-PRO-3.51
		304-THR-3.39
Anisotine	−8.23	5-LYS-3.31
		288-GLU-3.88
		291-PHE-3.15
Vasnetine	−8.78	05-LYS-3.4
		07-ALA-3.52
		126-TYR-3.37
Orientin	−6.06	03-PHE-2.88
		04-ARG-2.93
		05-LYS-2.78
		288-GLU-3.75
Ritonavir	−7.25	5-LYS-3.85
		126-TYR-3.28
		137-LYS-2.29
		284-SER-3.57
Nirmatrelvir	−8.10	5-LYS-3.40
		128-CYS-3.65
		291-PHE-3.42

## Data Availability

All the data originating from this research is available from the authors under request.
